#  Neural Differentiation of Human Umbilical Cord Mesenchymal Stem Cells by Cerebrospinal Fluid 

**Published:** 2015

**Authors:** Shirin FARIVAR, Zahra MOHAMADZADE, Reza SHIARI, Alireza FAHIMZAD

**Affiliations:** 1 Department of Genetics, Faculty of Biological Sciences, Shahid Beheshti University, Tehran, Iran; 2 Laser and Plasma Research Institute, Shahid Beheshti University, Tehran, Iran; 3 Department of Pediatrics, Shahid Beheshti University of Medical Sciences, Tehran, Iran; 4 Mofid Children’s Hospital, Pediatrics Infectious Research Center (PIRC), Shahid Beheshti University of Medical Sciences, Tehran, Iran

**Keywords:** Cerebrospinal fluid, Neurogenesis, Mesenchymal stem cells, Nestin, Microtubule-associated protein 2, Glial fibrillary astrocytic protein

## Abstract

**Objective:**

Wharton’s jelly (WJ) is the gelatinous connective tissue from the umbilical cord. It is composed of mesenchymal stem cells, collagen fibers, and proteoglycans. The stem cells in WJ have properties that are interesting for research. For example, they are simple to harvest by noninvasive methods, provide large numbers of cells without risk to the donor, the stem cell population may be expanded in vitro, cryogenically stored, thawed, genetically manipulated, and differentiated in vitro. In our study, we investigated the effect of human cerebrospinal fluid (CSF) on neural differentiation of human WJ stem cells.

**Material & Methods:**

The cells in passage 2 were induced into neural differentiation with different concentrations of human cerebrospinal fluid. Differentiation along with neural lineage was documented by expression of three neural markers: Nestin, Microtubule-Associated Protein 2 (MAP2), and Glial Fibrillary Astrocytic Protein (GFAP) for 21 days. The expression of the identified genes was confirmed by Reverse Transcriptase PCR (RT-PCR).

**Results:**

Treatment with 100 and 200μg/ml CSF resulted in the expression of GFAP and glial cells marker on days 14 and 21. The expression of neural-specific genes following CSF treatment was dose-dependent and time-dependent. Treatment of the cells with a twofold concentration of CSF, led to the expression of MAP2 on day 14 of induction. No expression of GFAP was detected before day 14 or MAP2 before day 21, which shows the importance of the treatment period. In the present study, expression analysis for the known neural markers: Nestin, GFAP, and MAP2 using RT-PCR were performed. The data demonstrated that CSF could play a role as a strong inducer.

**Conclusion:**

RT-PCR showed that cerebrospinal fluid promotes the expression of Nestin, MAP2, and GFAP mRNA in a dose-dependent manner, especially at a concentration of 200 μl/ml. In summary, CSF induces neurogenesis of WJ stem cells that encourages tissue engineering applications with these cells for treatments of neurodegenerative defects and traumatic brain injury.

## Introduction

Neural stem cells are immature, uncommitted cells in developing brains and an adult nervous system that can differentiate into neurons, astrocytes, and oligodendrocytes. However, neural stem cells from the central nervous system are rare, require an invasive procedure to obtain, and may have a more limited potential to proliferate in culture than embryonic or other adult stem cells. 

Mesenchymal stem cells or multipotent stromal cells (MSCs) are adult multipotent cells that have been isolated from almost all type of connective tissue .They have two main stem cells properties: self-renewal and ability of differentiation into mesodermal, endodermal, and even ectodermal lineages under appropriate in vitro conditions (2). 

MSCs appear to have a remarkable clinical potential in tissue regeneration and immune regulatory therapeutic applications. This source of stem cells was first identified by Friedenstein et al as fibroblast-like cells with clonogenic potential due to evoking only minimal immunoreactivity (3) and secretory of bio-active factors within- inflammatory and immuno-modulatory effects in vivo (4). To date, the best characterized source of MSCs is bone marrow (BM) and most of the knowledge regarding MSCs is based on BM studies. However, processing and harvesting of MSCs from BM has certain limitations as follows: a highly invasive procedure to the donors, significant reduction in number of MSCs per nucleated marrow cell with age, decline of differentiation potential, and proliferation efficiency of BM-MSCs with increasing age (5). 

Postnatal stem cells, such as mesenchymal stem cells as regards to their easy isolation and high proliferation as well as differentiation potential may offer a better source of cells for therapeutic purposes and neural repair. 

Brand new sources of MSCs and an alternative source is the umbilical cord that, as a fetus-derived tissue, can be easily obtained and processed and is noncontroversial (6). Wharton’s jelly (WJ) from umbilical cord possesses desirable characteristics such as a large, rapidly available donor pool, noninvasive and painless collection procedure, and ethically noncontroversial source of MSCs. WJ-MSCs have extensive in vitro expansion capabilities, wide multipotency, and do not induce teratomas(7). 

They are believed to be more primitive than MSCs derived from other tissue sources (8).Cerebrospinal fluid (CSF) circulates within the ventricles of the brain, and surrounds the brain and spinal column. The total volume of CSF in the human ventricular system is ca. 125 ml. CSF contains small molecules, salts, peptides, proteins, and enzymes, among others (9). The protein component of CSF consists of brain-derived proteins as well as many proteins that are also abundant in plasma (10). Recent studies suggest that CSF plays an important role in physiological as well as path physiological processes of the brain including adult neurogenesis (11, 12 and 13). 

Recent investigations concentrated mainly on proteins, membranous particles, amino acids, and on growth factors such as FGF2 as the components of CSF influencing neuroectodermal cell behavior (14, 15, 16, 17 and 18). In human CSF, many proteins with a known influence on cell survival, neural and glial differentiation, and proliferation and signal transduction were found (such as transthyretin, serine, retinol binding protein, heparin sulfate, several apo-lipoproteins, and FGF2) (19). 

In 2003, Mitchell et al described neural differentiation of human WJ using two main and well-known neural differentiation inducers: DMSO and BHA(20).We present our findings as to the effect of CSF, a human body fluid containing different growth factors, on neural differentiation of human WJ MSCs evaluating three neural marker expression: MAP2, Nestin, and GFAP. 

## Materials & Methods

Wharton’s jelly stem cell culture 

**Fig 1:**
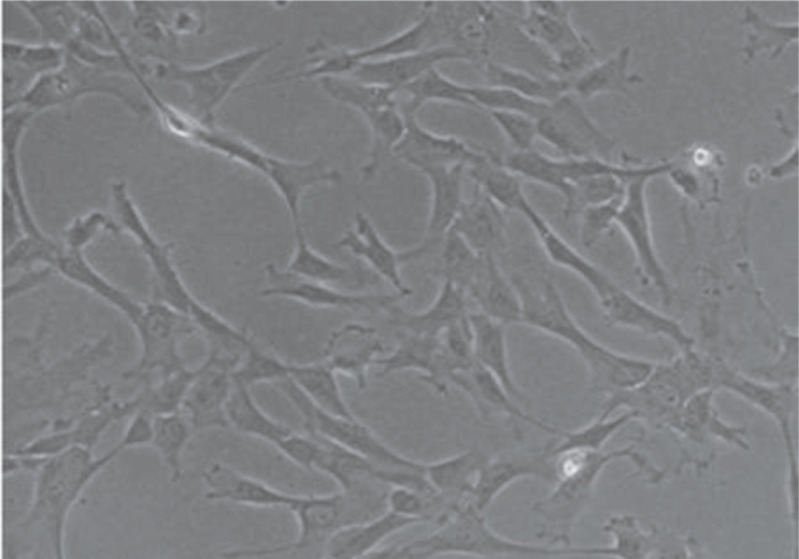
Morphology of passage 3 WJ-MSCs cultured in DMEM medium containing 10% FBS (x40)

Human WJ stem cells (passage 2; normal cerotype) were obtained from Shahid Beheshti University of Medical Sciences. Human WJ stem cells were Isolated from fresh human umbilical cords. Cells were kept in an undifferentiated state in Dulbecco’s Modified Eagle Medium (DMEM) (Invitrogen) supplemented with 10% fetal bovine serum (FBS; Gibco) and 1% pen-strepto (Gibco) and medium exchange was done every 2 days (Fig1). Reaching 80% confluence, the adherent cells were detached with 0.05% trypsin-EDTA, counted with Trypan Blue exclusion, and reseeded at 3000 cells/cm2 to reach the 90% of confluence after 3-4 population doublings. 

Normal child CSF was provided after informed consent from Mofid Children’s Hospital. Collected CSF was transferred to a sterile microtube on ice and centrifuged (Hettich lab technology universal, 320R, Germany) at 8000 rpm for 10 min to remove cells or debris, and then supernatants were passed through 0.45-μm and 0.22-μm filters sequentially and were stored at −20°C until use. 

**Differentiation of Human Wharton’s jelly stem cells**

Cells were seeded into T25 cell culture flasks in the presence of DMEM-F12 medium supplemented with 1% FBS and 1% penicillin-streptomycin. Neural differentiation of cells was induced by culturing the cells in the medium containing the collected CSF as following. Before treatment, the cells were divided into 7 groups and each group contained cells treated with human CSF at different periods of time as follows: in group A the cells were treated with CSF for 1day; in group B for 4days; in group C for 7days; in group D for 14days and in group E the cells were treated with CSF for 21days; in group F the cells were treated with all trans Retinoic acid (10μl/ml); and in group G the cells were cultured in only DMEM-F12 media supplemented with 1% FBS. Additionally, group(s) F and G was considered as positive and negative controls, respectively. Each group was subdivided into two subgroups and two concentrations (100μl and 200 μl per ml) of CSF were added to each subgroup. The whole process was performed in triplicate. 

**Cell viability**

The addition of any new factors into the cell environment will affect the faith of the cells. The cytotoxicity of added factors depends on the additional dose. Different studies have shown that even a high dose of an inducer might have a cytotoxic effect on the cells. We stained with Trypan Blue after each harvest (before RNA extraction) and the percentage of viable cells was evaluated using hemocytometer lam to investigate cell viability and the effect of human CSF as an exogenous protein cocktail and the dosages on cell viability. 

**RNA extraction and cDNA synthesis**

RNA extraction was performed at days 1, 4,7,14, and 21 of differentiation after initial cell seeding. The expression of neural markers, microtubule-associated protein 2 [Map2] as a mature neuron marker), Nestin (as an immature neuron marker) and (Glial fibrillary acidic protein [GFAP] as mature astrocyte marker), was assessed by Reverse Transcriptase-PCR (RT-PCR). 

Total RNA was extracted using an Easy Blue Total RNA Extraction Kit according to the manufacturer’s instructions. Samples were treated with RNase-free DNase I (Takara) to avoid DNA contamination. Extracted RNA concentration was measured by a NanoDrop™ 2000 (Spectrophotometers; Thermo Scientific, USA) and cDNA was synthesized from 1μg of RNA using cDNA Reverse Transcription Kit (Takara) according to the manufacturer’s instructions. 

The samples were incubated at 42°C for 60 min, followed by 10 min incubation at 72°C. Polymerase chain reaction (PCR) was carried out using standard protocols with Taq DNA Polymerase (Gibco-BRL). PCR conditions included a first step of 3 minutes at 94°C, a second step of 25–30 cycles of 30 seconds at 94°C, a 45-second annealing step at 53–62°C, 1 minute at 72°C, and a final step of 10 minutes at 72°C. The expression value of each gene was normalized to the amount of ß-Actin cDNA to calculate a relative amount of RNA present in each sample. The PCRs were performed in triplicate. Table 1 provides details of the primers and amplicon size. The final products were examined by gel electrophoresis on 1.5%agarose ethidium bromide-stained gels. 

**Imaging and Statistical analysis**

Images were obtained with the Olympus Stereomicroscope and invert microscope. The data were stated as mean ± SD (standard deviation) on version 16 of SPSS statistical software. Statistical significance between groups was calculated by independent Student t test assuming equal variance. P <0.05 was considered statistically significant. 

## Results

**Fig 2:**
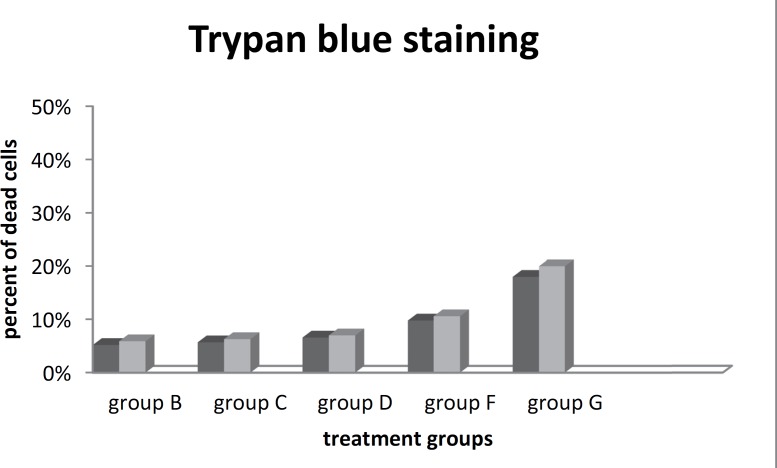
Cell viability diagram

Our initial interest was to determine whether new factors into the cell environment will affect the faith of the cells. Trypan blue cell staining showed that there was no significant cell death after treating cells with CSF (near 10%) when compared to routine neural differentiation factor retinoic acid (18%). In addition, our results demonstrate that no significant differences in cell death were observed between the two concentrations of inducer. Data are expressed as mean ±SD. For comparison of quantitative measures, the values were subjected to statistical analysis by using the Student’s t-test and were considered significant with p <0.05 (Fig2). 

**Fig 3:**
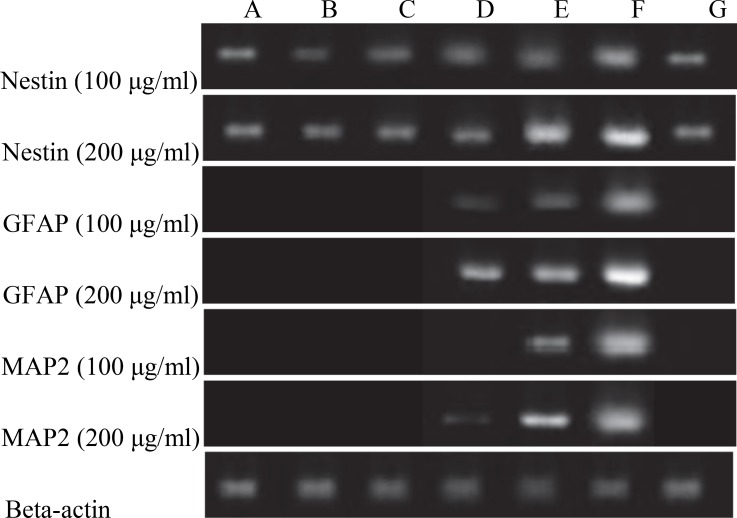
RT-PCR results, A: day 1, B: day 4, C: day 7, D: day 14, E: day 21, F: RA treatment 10μM, G: negative control

The influence of CSF on characteristics of isolated WJ stem cells was investigated with RT- PCR. In order to evaluate effect of CSF on neural gene expression in WJ-derived MScs, neural gene expression was assessed and compared in all groups. Three established transcripts, Nestin, MAP-2, and GFAP were evaluated in cells after cultivation. Previous studies had shown that human WJ stem cells are Nestin positive cells but in our study, treatment with CSF exhibited a remarkable increase in expression of Nestin during 21 days of treatment. Treatment with 100 and 200 μg/ml CSF resulted in the expression of GFAP, the glial cells marker, on days 14 and 21, respectively; when compared with the cells cultured in the control groups. MAP2, the marker of neural differentiation, showed a delayed expression on day 21 of treatment with100 μg/ml CSF. GFAP is expressed before MAP2 at 100ug/ml concentration. On Day 14, at 200ug/ml CSF concentration, GFAP expression is higher than MAP2 expression (Fig3). The expression of neural-specific genes that occurred following CSF treatment was found to be dose-dependent and time-dependent. Treatment of the cells with a twofold concentration of CSF led to expression of MAP2 on day 14 of induction to demonstrate the effect of inducer dose on the differentiation of stem cells. Figure 2 shows that no expression of GFAP was detected before day 14 or MAP2 before day 21 to show the importance of treatment period. In the present study, expression analysis for the known neural markers Nestin, GFAP, and MAP2 using RT-PCR was performed and the data demonstrated that CSF could play a role as a strong inducer. 

## Discussion

The potential for self-renewal and multi lineage differentiation indicate that MSCs are an attractive therapeutic tool. Several reports have indicated that extra embryonic tissues might be considered a potential source for populations of cells that phenotypically resemble BM-MSC (21). This has been demonstrated for the placenta (22), amniotic fluid (23), amniotic membrane (24), umbilical cord blood (25), and umbilical cord matrix (26). 

MSCs isolated from WJ and bone marrow are shown to have an inherent potential to differentiate along the mesodermal lineage such as adipogenic, chondrogenic and osteogenic cells (27). Many reports have shown that WJ is an alternative source for human MSCs for experimental and clinical applications in comparison with BM (28). 

A number of neurodegenerative diseases result in cell death. Cell-based therapies involving stem cells aim to replace these lost cells or repair damaged areas, thus providing functional recovery. However, before the potential of stem cell-based therapies can be realized, it is important to understand the behaviour of these cells after implantation in vivo (29). Stem cells derived from WJ have a utility in autologous stem cell therapy; they not only reduce the need for immunosuppression but are harvested relatively easily in the clinic. In the present study, we investigate the potential of human CSF on neural differentiation of human WJ stem cells. We report a novel method to modulate the differentiation of human WJ stem cells into neurons involving the use of Normal child CSF. WJ-MSCs from the umbilical cord are of epiblast origin and show multipotent characteristics between embryonic stem cells and adult stem cells. They have higher proliferation rates and a lower tendency to differentiate into adipocytes when compared to bone marrow MSCs (30). Moreover, WJ-MSCs can replicate through many passages without karyotypic changes or senescence; and they can retain stemness properties for a long time in-vitro. 

Weiss et al (31) demonstrated the differentiation capacity of human umbilical cord mesenchymal stem cells into dopaminergic neurons. Most recently, Carlin et al (32) demonstrated some embryonic stem cell markers, such as Oct-4, Sox-2, and Nanog for the first time in porcine umbilical cord matrix cells. Through many passages without karyotypic changes, they are similar to bone marrow stromal and other MSCs in their surface marker CD 13, CD 29, CD 44, CD 73, CD 90, and CD 105; and negative for CD 34, CD 45, CD 14, CD 33, and HLA-DR (33). 

**Table 1:** Primers Sequences

Product size (bp)	Tann (ºC)	Antisense primer	Sense primer	Gene
172	58 ºC	5'-CCCACTTTCTTCCTCATCTG -3'	5'-CTCTGACCTGTCAGAAGAAT-3'	Nestin
180	53 ºC	5'- CCTGCTTGCCGGGCTCACCCGA-3'	5'-CCAATGGATTCCCATACAGG-3'	Map2
165	53 ºC	5'- CCTCCGTGTAGTGACCCTTG-3'	5'- AACGAGGCCTCTTCTCACAA-3'	GFAP
357	62 ºC	5'- AGTCCGCCTAGAAGCATTTG-3'	5'- CACTCTTCCAGCCTTCCTTC-3'	ß-actin

We have demonstrated that human CSF, a normal body fluid, has a noteworthy potential to induce differentiation in vitro culture. In this study expression of Nestin, GFAP, and MAP2 markers was significantly increased compare to with the cells cultured in the control groups (p<0.001). 

The system used in this study has the advantage that human CSF contains several growth factors such as bFGF or EGF along with other factors that promote and enhance the development of neural cells apart from glial induction and natural neural inducers. In this study, even high concentration of human CSF, has shown a low cytotoxic effect on cells compared with other well-known inducers. In summary, differentiation can be induced by human CSF in a dose dependent manner. 

Thus it seems the WJ MSC-derived neurons can be used for treating neurodegenerative disease and can be easily obtained from discarded cord blood.
